# Syringe services program staff and participant perspectives on changing drug consumption behaviors in response to xylazine adulteration

**DOI:** 10.1186/s12954-024-01082-y

**Published:** 2024-08-30

**Authors:** William H. Eger, Marina Plesons, Tyler S. Bartholomew, Angela R. Bazzi, Maia H. Hauschild, Corbin C. McElrath, Cyrus Owens, David W. Forrest, Hansel E. Tookes, Erika L. Crable

**Affiliations:** 1 School of Medicine, University of California San Diego, La Jolla, CA, USA; 2 School of Social Work, San Diego State University, San Diego, CA USA; 3 Department of Medicine, Miller School of Medicine, University of Miami, Miami, FL USA; 4 Department of Public Health Sciences, Miller School of Medicine, University of Miami, Miami, FL USA; 5 Herbert Wertheim School of Public Health, University of California San Diego, La Jolla, CA, USA; 6 Boston University School of Public Health, Boston, MA USA; 7 Present address: 9500 Gilman Dr, La Jolla, 92093 CA, USA

**Keywords:** Xylazine, Opioids, Injection drug use, Harm reduction, Drug supply, Overdose

## Abstract

**Background:**

Xylazine is an increasingly common adulterant in the North American unregulated drug supply that is associated with adverse health outcomes (e.g., skin infections, overdose). However, there are significant knowledge gaps regarding how xylazine was initially identified and how syringe services program (SSP) staff and clients (people who use drugs) responded to its emergence.

**Methods:**

From June–July 2023, we conducted qualitative interviews with medical (e.g., clinicians) and frontline SSP staff (e.g., outreach workers) and adult clients with a history of injection drug use at a Miami-based SSP. Inductive memos identified emergent codes; thematic analysis involving team consensus established final themes.

**Results:**

From interviews with SSP staff (*n* = 8) and clients (*n* = 17), xylazine emergence was identified at different times, in various ways. Initially, during summer 2022, clients identified a “tranquilizer-like substance” that worsened sedation and withdrawal and caused wounds. SSP medical staff later identified this adulterant as xylazine by treating new medical cases and through diverse information-sharing networks that included professional societies and news sources; however, frontline SSP staff and clients needed additional educational resources about xylazine and its side effects. With limited guidance on how to reduce harm from xylazine, SSP clients altered their drug consumption routes, reduced drug use, and relied on peers’ experiences with the drug supply to protect themselves. Some individuals also reported preferring xylazine-adulterated opioids and increasing their drug use, including the use of stimulants to avoid over sedation.

**Conclusions:**

Xylazine’s emergence characterizes the current era of unprecedented shifts in the unregulated drug supply. We found that xylazine spurred important behavioral changes among people who use drugs (e.g., transitioning from injecting to smoking). Incorporating these experiences into early drug warning surveillance systems and scaling up drug-checking services and safer smoking supply distribution could help mitigate significant health harms caused by xylazine and other emergent adulterants.

**Supplementary Information:**

The online version contains supplementary material available at 10.1186/s12954-024-01082-y.

## Background

The prevalence of illicitly manufactured synthetic substances is on the rise globally [1–4]. In North America, the ongoing overdose crisis is driven by an increasingly toxic unregulated drug supply that includes illicitly manufactured fentanyls (IMFs) and other novel synthetic substances like xylazine [5–7], a potent sedative hypnotic alpha-2 adrenergic agonist approved as a veterinary tranquilizer [8]. However, since 2021, its presence has proliferated in the unregulated opioid supply, being detected in every major U.S. census region [5, 9]. Xylazine has significant health implications for people who use drugs (PWUD), given its association with heightened sedation and severe tissue necrosis that can produce skin and soft tissue infections (SSTIs) [8, 10–12]. In some U.S. jurisdictions, xylazine has been involved in more than 30% of overdose deaths involving IMFs [5, 13].

While systematic surveillance of xylazine is still developing, U.S. jurisdictions with the capacity to measure xylazine are reporting rapidly increasing prevalence levels, especially in the Southern U.S. [5, 9, 14, 15]. For instance, between 2021 and 2023, 39% of overdose deaths involved xylazine in Jefferson County, Alabama [14], compared to just 8% in 2021 [5]. In Miami-Dade County, Florida, overdoses involving xylazine and IMFs increased sixfold between 2019 (4%) and 2021 (24%) [15]. Yet, outside of xylazine prevalence data, no studies to our knowledge have examined experiences of xylazine use among PWUD in the Southern U.S.

In response to rising xylazine adulteration in Miami, a local syringe services program (SSP) began using xylazine test strips (XTS) in April 2023. Due to state law prohibiting the distribution of XTS or testing of xylazine adulterated supplies directly [16], the SSP used XTS in urine samples obtained from clients, detecting xylazine in 56% of samples from clients reporting recent IMF use between April and August 2023 [17]. In the face of this evidence, researchers affiliated with the SSP aimed to explore experiences with xylazine’s emergence and opportunities for additional intervention among SSP staff and PWUD. This research also aimed to characterize the potential impacts of xylazine’s presence in the drug supply on drug consumption and social behaviors of PWUD.

## Materials and methods

### Setting, design, and population

This study was conducted at the IDEA Miami SSP in Miami, Florida, which operates through a fixed site and mobile unit. This site was chosen for recruitment because SSP medical providers had recently identified xylazine in their drug supply amid rising xylazine adulteration in the Southern U.S., and the SSP was in the midst of implementing XTS [17]. This provided an opportune scenario to understand the emergence of xylazine from the perspective of SSP staff and PWUD. In addition to the distribution of harm reduction supplies (e.g., syringes; naloxone; fentanyl test strips), the SSP offers healthcare services, including treatment for opioid use disorder (e.g., buprenorphine), prevention and treatment of HIV, basic clinical services (e.g., phlebotomy; wound care), and facilitates referral to social services.

The first author (WHE), a researcher trained in qualitative methods and harm reduction service delivery, recruited SSP staff, including medical providers (e.g., clinicians) and frontline SSP staff (e.g., outreach workers) to participate in interviews via email or in-person communications. Staff were not compensated for their involvement.

WHE also recruited adults (≥ 18 years old) with a history of injection drug use who were clients actively seeking services from the SSP (e.g., while exchanging syringes or after their medical appointment). Participants were selected using purposive sampling to ensure the inclusion of SSP staff and clients who had direct experiences with the identification and impact of xylazine in the unregulated drug supply. All interviewees completed a brief verbal informed consent process, and clients were compensated $20 cash for their time. All study procedures were approved by the University of Miami Institutional Review Board.

### Data collection

Semi-structured interviews with SSP staff and clients were conducted between June and July 2023, soon after the identification of xylazine in the unregulated opioid supply in Miami and the SSP began altering services to address the new substance [17, 18].

The research team developed two semi-structured interview guides (see Appendices 1 and 2); one tailored to SSP staff and another to client experiences. Both interview guides aimed to identify opportunities to support SSPs in providing drug checking (e.g., urine testing, XTS) and other xylazine-related services (e.g., wound care). Primary domains for SSP staff interviews included (1) xylazine-related knowledge and experiences, (2) knowledge and experiences with drug-checking services, generally, (3) knowledge and experiences with xylazine-specific drug-checking services, and (4) perceived changes to participants’ substance use. Client interviews covered three primary domains: (1) experiences with xylazine, (2) perceptions of xylazine drug testing, and (3) personal and perceived changes to substance use due to xylazine.

SSP staff participated in individual interviews in person or via Zoom, depending on their preference, while all client interviews were conducted by the first author in person, in confidential rooms at the SSP. The first author and DWF had weekly discussions to monitor for thematic saturation to ensure interviews were capturing a breadth and depth of information related to our key interview domains. SSP staff interviews lasted between 28 and 70 min, and client interviews ranged between 20 and 50 min. Interviews were recorded, professionally transcribed, and reviewed by the research team for accuracy (WHE, MHH, CO).

### Analysis

Four qualitative analysts (WHE, ELC, MHH, CCM) used “open coding” to allow inductive themes to emerge from the data [19, 20]. The analysts first developed qualitative memos from four transcripts (two SSP staff and two client) to develop potential codebook codes, definitions, and inclusion and exclusion criteria. The qualitative research team refined the codebook through consensus discussions. To ensure rigorous and consistent application of the codes, two qualitative researchers (WHE, ELC) double-coded 20% of the transcripts. Conflicts were addressed through discussion and resolved by refining and retesting the codebook until a final version was developed. After establishing coding consensus, one researcher (WHE) coded the remaining transcripts. Two qualitative researchers (WHE, ELC) reviewed all coded passages to develop summary statements about potential themes and the qualitative research team refined the final themes through consensus discussions. All qualitative analyses were conducted in NVivo (QSR International Pty Ltd, 2020).

## Results

***Participant Characteristics.*** We achieved thematic saturation in response to our guiding questions after conducting 25 interviews, which included eight SSP staff members, consisting of four medical providers (two physicians, one nurse practitioner, and one medical assistant) and four frontline staff (one outreach coordinator, one social worker, one community resources coordinator, and one medical student volunteer) and 17 SSP clients with a history of injection drug use. Table 1 describes characteristics (e.g., socio-demographics, drug use behaviors) of SSP clients who were interviewed.

**Table 1:** Characteristics of syringe services program clients (*n* = 17)

Participant characteristic^*^	*n* (%)^a, b^
**Median age in years (IQR)**	42.5 (9.0)
**Race**	
White	16 (94.1)
Black/African American	1 (5.9)
**Hispanic**	6 (35.3)
**Male**	14 (82.4)
**Highest level of education**	
Some college or higher	9 (52.9)
High school diploma or equivalent	6 (35.3)
Less than high school	1 (5.9)
Unknown	1 (5.9)
**Employment status**	
Unemployed	12 (70.6)
Employed	4 (23.5)
Unknown	1 (5.9)
**Experiencing homelessness**	10 (58.8)
**Drugs injected (past 30 days)** ^**c**^	
Heroin or fentanyl	16 (94.1)
Methamphetamine	4 (23.5)
Crack/cocaine	14 (82.4)
Other	1 (5.9)
**Drugs smoked (past 30 days)** ^**c**^	
Heroin or fentanyl	3 (17.6)
Methamphetamine	2 (11.8)
Crack/cocaine	7 (41.2)
Other	6 (35.3)

^*^ Characteristics were assessed at program enrollment and may not express current behaviors

^a^ Values are n (%) for categorical variables and median for continuous variables

^b^ Numbers may not sum to total due to missing data, and percentages may not sum to 100% due to rounding

^c^ Participants could select all that apply

Abbreviations: IQR = interquartile range

***Qualitative Themes***. We identified six themes (Table 2) organized in response to two guiding research questions: (1) how information about xylazine adulteration spread, and (2) how SSP clients altered their behavior in response to identifying this new adulterant in the drug supply.

**Table 2:** Qualitative themes

**Guiding Question 1: How did information about xylazine adulteration spread?**
**Theme 1**: Clients initially identified a tranquilizer-like adulterant that heightened sedation and withdrawal symptoms and caused wounds.
**Theme 2**: SSP medical providers identified xylazine by treating new medical cases and through diverse information-sharing networks including professional societies and news sources. • *Medical cases*: wounds in non-injection sites, tissue necrosis, challenges alleviating withdrawal symptoms • *Information sharing networks*: professional societies, conferences, harm reduction partners, media coverage
**Theme 3**: SSP frontline staff and clients needed additional educational resources about xylazine and its potential side effects. • *Desired educational materials*: staff training, client-facing pamphlets
**Guiding Question 2: How did SSP clients respond to seeing xylazine in the drug supply?**
**Theme 4**: Clients began altering their drug consumption routes, reducing drug use, and relying on their peers’ experiences with the drug supply to protect themselves from xylazine. • Transitioning from injecting to smoking, anal administration • Using the same seller, ‘street science’ testing methods, sharing safe supplies
**Theme 5**: Xylazine’s emergence led some individuals to prefer xylazine-adulterated opioids and to increase their drug use. • Potentially prolonged, intense high, conflicting perceptions of xylazine’s half-life
**Theme 6**: Seemingly protective behaviors like increasing the use of stimulants, using alone, and conducting ‘human testing’ placed clients in harm’s way. • Mixing drugs, using alone, ‘human testing’ for potency

**Note**: SSP = syringe services program.

### Question 1. How did information about xylazine adulteration spread?

Xylazine was often described as “*tranq*” or “*tranq dope*”, especially by clients, who noted that “*people call it tranq. Nobody really calls it xylazine.*” One frontline SSP staff member recounted they “*never heard a participant describe it as xylazine*,* always as tranq.”* Other terms commonly used to describe xylazine in the unregulated opioid supply were “*knockout dope*” and “*anestesia de caballo*” (‘horse tranquilizer’).

The emergence of xylazine in the unregulated opioid supply in Miami was identified at different times, in various ways, by clients, medical providers and frontline SSP staff. Clients were the first to notice a shift in the drug supply in late summer 2022. Medical providers and frontline SSP staff later identified the adulterant as xylazine in late winter/early spring 2023.

#### Theme 1: clients initially identified a tranquilizer-like adulterant that heightened sedation and withdrawal symptoms and caused wounds

The first signs of xylazine in Miami occurred when clients noticed an unidentified adulterant in the unregulated opioid supply that produced rapid, tranquilizer-like sedation that was unlike fentanyl. One client recalled a common experience amongst their peers: “*The first time I was told about it was after falling asleep multiple times and getting robbed for all my belongings. I couldn’t understand why I kept passing out. People around me were telling me that*,* “Yeah*,* there’s tranq in it.*”

Some clients initially thought the new substance *“was just good fentanyl”* that produced more intense sedative effects. However, clients then began experiencing more rapid onset of withdrawal symptoms as one client said, “*Too many people were complaining that they were gettin’ sick from the dope - not like a normal sickness*,*”* suggesting an adulterant in the drug supply. Clients said they and their peers developed more severe and necrotizing skin and soft tissue infections to the extent that “*a lot of people… just have open wounds all over their body.*” By spring 2023, clients said the heightened sedation, rapid onset of withdrawal symptoms, and wounds were common, and they attributed these symptoms to an unidentified type of “*tranq*” that was ubiquitous in the unregulated opioid supply.

#### Theme 2: SSP medical providers identified xylazine by treating new medical cases and through diverse information-sharing networks including professional societies and news sources

While clients were aware of a tranquilizer-like adulterant in the drug supply, the SSP’s medical providers were the first to identify it as xylazine. Their suspicions were raised when clients *“started showing up with wounds in non-injection site-related areas*” and “*were having difficult[y] just getting [their wounds] healed.*” One provider recalled a case when a client’s “*fingers auto amputated*,* so she’s missing the end of her digits*,* the majority of them. Then after that*,* we just started to see more and more of these necrotic wounds on our participants.”* This case exemplified the growing number of atypical wounds (e.g., those occurring at non-injection sites and demonstrating tissue necrosis) providers began treating among the SSP’s clients. Medical providers also noticed new challenges in effectively treating opioid use disorder, especially in managing their clients’ withdrawal symptoms while they transitioned onto medications for opioid use disorder. As one provider noted, some clients experienced *“really extreme anxiety”* when initiating buprenorphine.

Meanwhile, all frontline SSP staff recalled “*hear[ing] a lot of secondhand reports and… anecdotal evidence of folks interacting with ‘tranq’ and it affecting them differently than fentanyl.”* One frontline staff member said an SSP client was hit by a car because of the sedative effects of the new adulterant: “*he just literally passed out on the street and didn’t know how it happened*,*”* which was *“not usually something we saw with just fentanyl or just regular dope.”* While frontline staff began to suspect a change in the unregulated supply, they “*didn’t have the institutional response. [They] didn’t have anything to test”* to identify the new adulterant.

In the fall of 2022, the SSP’s medical providers began receiving information about xylazine from their professional societies and harm reduction partners. For example, one healthcare provider attended a medical conference and learned about a case report in which mass spectrometry was used to confirm a xylazine-related wound. When an SSP client presented with severe tissue necrosis later that winter, the healthcare provider remarked that the case *“was identical to [the client’s] wound*,* in every way.”* This provider then educated their colleagues who were unaware of the term ‘xylazine’; they recalled, *“I remember mentioning it on an… [SSP team] call*,* and nobody had heard of it.”* Few frontline staff said they became aware of xylazine through the media or other harm reduction information networks; rather, they mostly learned about it from the SSP’s medical providers.

Their collective awareness of xylazine subsequently grew through knowledge-sharing networks of statewide harm reduction organizations and increasing coverage of xylazine in the media. One medical provider described how the emergence of any new substance represents *“a bunch of unknowns”* and that alone, they would *“have no idea what to do.”* However, by combining medical providers’ pharmacology knowledge and harm reductionists’ knowledge of the drug use community, they were collectively able to identify the substance as xylazine and share information about its emergence in the unregulated supply.

Once the new adulterant was hypothesized to be xylazine in late winter 2022, the SSP’s medical providers ordered XTS. The SSP’s use of XTS in spring 2023, approximately six months after clients began noticing the widespread proliferation of a tranquilizer-like substance in the unregulated opioid supply, officially enabled the SSP to classify “*tranq*” as xylazine.

#### Theme 3: SSP frontline staff and clients needed additional educational resources about xylazine and its potential side effects

SSP frontline staff then started educating clients about xylazine. Although SSP clients were already “*aware that something [was] different*,*”* SSP staff were able to provide information about the adulterant and its potential side effects. One frontline staff member said, *“Now they’re gaining the knowledge of*,* ‘Oh*,* wait*,* this can cause more problems than I thought.’”*.

At the time of data collection, many interviewees, including some frontline SSP staff, said they needed more education about xylazine. One medical provider said, “*We don’t have a lot of the answers that we can provide to the patients.”* Some frontline staff said they were going to self-study to “*try to learn more about xylazine*,* ‘cause I think it looks pretty bad if I have a conversation with a participant*,* and they bring up xylazine to me and I’m just clueless.*” Frontline staff said they needed more educational materials about xylazine from the medical and harm reduction communities; “*If we had just a pamphlet or something that we can also provide to the participants for them to have an idea of what they should do*,* or for all of us to be trained in what we should expect*,* that’d be very nice.*”

### Question 2. How did SSP clients respond to seeing xylazine in the drug supply?

In the context of little formal evidence on how to respond to (or protect themselves from) xylazine, clients developed strategies to protect themselves from its side effects based on their own experiential knowledge. Clients described altering their drug use behaviors in various ways, including some that are indeed protective and some that are seemingly protective but carry potential adverse health consequences.

#### Theme 4: clients began altering their drug consumption routes, reducing drug use, and relying on their peers’ experiences with the drug supply to protect themselves from xylazine

The most common behavior clients used to protect themselves from rising xylazine adulteration was transitioning from injecting to smoking opioids. Some clients also described using other alternative consumption routes, like anal administration using a syringe (without the needle) to avoid injecting, snorting, or smoking. Clients said they reduced their injection drug use for four main reasons: (1) to avoid wounds or other adverse health consequences (e.g., overdose) reported to be worsened by xylazine; (2) their veins had become inaccessible or they were concerned about their veins becoming inaccessible; (3) they felt withdrawal symptoms more quickly than with fentanyl alone and did not want to inject as frequently to curb withdrawal; and (4) they generally perceived non-injection consumption routes as safer than injecting. Additional details about these drug consumption behaviors are described below.

SSP staff and clients said they noticed an increase in non-injection consumption routes since the emergence of xylazine, but it was not always clear why this transition occurred. Some interviewees attributed the transition directly to experiences with xylazine. One medical provider said they had *“a handful of patients that are now more afraid to inject and are starting to smoke it more or they’re doing maybe bumping or things like that.”* This medical provider rationalized that *“these are patients that have probably had bad experiences with wounds that are starting to reconsider injection.”* Clients generally attributed the change in their route preference to overall declining vein health resulting from an increasingly toxic drug supply that included xylazine: *“I started smoking it. That came around with the tranq. I don’t know why*,* if it’s from the tranq or if it’s from the Molly fucking up my veins… I know a lot of people that smoke the tranq dope.”*

Interviewees also described that some clients had reduced or intended to reduce the use of unregulated supplies because they did not like the sedative effects of xylazine. Additionally, some clients said they were worried about the unintended consequences of xylazine, including tissue necrosis or that naloxone would not be able to reverse a xylazine-involved overdose. Medical providers noted that a growing desire to reduce or stop the use of unregulated opioids represented an opportunity to support more clients in their recovery journey with medications for opioid use disorder. As one medical provider said, “*I have a general feeling that there’s more people who are like*,* I really want to try to avoid that. Perhaps now is a good time to get onto bup[renorphine]*,*”* or help counsel patients on how to decrease substance use-related harm.

Clients also reported consistently buying their drugs from a supplier they trusted as a protective behavior to ensure they are using familiar supplies. When clients had to go to a new supplier, they often used smaller or the same amounts, or they had peers test it for them either through XTS given by the SSP or via (what we defined as) ‘street science’ methods. Clients also described ‘street science’ approaches to assess the safety of a new drug supply, including conducting ‘human testing’ (i.e., watching someone use a new supply or asking them about their experience), using a black light, or visually inspecting the color of their drugs (both before and after combustion). One client described how fentanyl *“glows with UV lights…and tranq doesn’t*,*”* stating that *“depending on how bright it glows”* they can tell *“how much fentanyl’s in it.”* Clients also described investigating how the color of their drugs changed with heat, suggesting whether opioids are adulterated with xylazine; clients described that xylazine turns black after combustion while fentanyl does not discolor.

Most clients said they preferred to use drugs that reportedly (through their peer network or ‘street science’ approaches) did not have xylazine or tested negative for xylazine. A few said they would attempt to return adulterated supplies to the person who sold them after seeing that their supplies tested positive for xylazine. One client rationed, “*maybe the dealer doesn’t know what’s in it. Maybe you gotta tell him to go get it checked himself.”*

Clients heavily relied on their peers to protect them from rising xylazine adulteration. For example, clients said they carried naloxone and test strips to share with others and hung around peers who used the same substances as them so they would have harm reduction supplies and could share information. Some clients described telling and/or showing their peers that a new supply was safe, highlighting that *“word of mouth—is huge*,*”* within their community, where some people often solely rely on *“word of mouth… [to] decide on it*,* who we were buying from.”* However, most clients regarded their peer’s opinions as more trustworthy than those of suppliers, “*because*,* if you ask the dope boy or dealer that you’re getting it from*,* they’re gonna lie to you no matter what. “Oh*,* no it doesn’t have it*,*” or “It does have it*,*” whatever they know you’re trying to do.”*

#### Theme 5: Xylazine’s emergence led some individuals to prefer xylazine-adulterated opioids and to increase their drug use

Numerous clients said they or someone they knew were seeking out xylazine. Interest in xylazine appeared to be a relatively “*new phenomenon*,* because initially everybody was like*,* “What? What is that? I’m not taking that.”* Reasons for seeking xylazine-adulterated opioids included 1) liking its “*sedative*,* calming effects*,*”* 2) wanting the more intense and immediate high xylazine provides compared to fentanyl, and 3) wanting the longer-lasting high from xylazine-adulterated fentanyl compared to fentanyl alone. One client explained, *“When it’s just fentanyl… I don’t get what I’m looking for entirely. If it’s just tranq*,* then I get nothing I want.”* They said, *“The two together*,* if they’re in the right ratio*,*”* provided the ideal high.

Although many clients said they decreased their drug use due to xylazine’s emergence in the supply, some noted that they or their peers were using drugs (predominantly fentanyl) more frequently due to xylazine adulteration. Some clients, particularly those who were interested in xylazine, reported nearly doubling the number of times they use opioids per day since xylazine entered the drug supply. Some clients also attributed the increase in use to the more rapid onset of withdrawal symptoms with xylazine-adulterated fentanyl. As one client explained, “*Most people are seeking tranq*,* I think*,* [because] it lasts so much longer*,* but at the same time*,* it’s not really getting your sick off.”* This trend was corroborated by frontline SSP staff: “*The people that are seeking tranq’ – their amount of bags that they buy has gone up. It’s congruent with what people are saying*,* that it doesn’t last as long… They’ve doubled*,* in a day*,* of what they’re using.”*

#### Theme 6: seemingly protective behaviors like increasing the use of stimulants, using alone, and conducting ‘human testing’ placed clients in harm’s way

Clients also employed behaviors intended to protect themselves as they encountered xylazine in the unregulated supply, but some of these new behaviors carried risks for unintended harm. First, many clients reported increasing their use of stimulants, “*speedball*,” or “*molly*” to prevent the intense sedative effects of xylazine that put them at risk of being robbed, physically and sexually assaulted. One client said, *“I think it’s actually a dumb idea to do any tranq without an upper [stimulant] with it.”*

Second, some clients said they started using drugs alone or in more secure areas where there were no bystanders who could rob or physically or sexually assault them while they were sedated by xylazine. One client said they started using “*alone because when I use with friends*,* and it’s got tranq in it*,* it knocks me out. They say I’m just out of control when I’m blacked out*.”

Lastly, as mentioned above, clients also said that ‘human testing’ of supplies helped them generate real-time information about the potency or safety of potential xylazine-adulterated supplies. Some people volunteer to use before their peers to show how they react to the drug and help others determine how much they should use, such as one client describing his peer: *“I’ll watch him if he shoots a shot*,* and then [if] he fuckin’ just straight nods out*,* I can tell it’s mostly tranq.”*

## Discussion

The emergence of xylazine has led to several unintended consequences for PWUD across North America, such as tissue necrosis and heightened sedation, the latter of which has potential implications for elevated risk of overdose and social consequences (such as being robbed or assaulted) [5, 21]. Here, we identified how the emergence of xylazine has also precipitated a range of behavioral responses among PWUD, with implications for their individual health and the broader healthcare system [22].

We identified dynamic communication processes through which information about xylazine emerged and spread among SSP staff and clients, ultimately leading to a formal response by the SSP via the use of XTS [17]. Notably, the initial identification of xylazine was driven by clients’ firsthand experiences with the unregulated opioid supply. Clients then shared their observations and experiences with SSP staff, underscoring the importance of building trust and listening to client expertise, which can serve as an early warning system for emerging adulterants prior to adverse consequences. In contrast, many of the official accounts of xylazine adulteration are based on overdose surveillance data or medical examiner reports [9, 13–15, 23], which can take time to process. In addition, most official early warning systems focus on mass spectrometry or drug seizure data, which may involve a time lag and is resource intensive [24, 25], especially in the Southern U.S. where comprehensive drug-checking services are sparse [26, 27]. Integrating qualitative insights from PWUD into current early warning systems may provide another avenue for proactive intervention that can indicate the presence of a novel adulterant using relatively few resources and can be scaled locally via SSPs or other settings that serve PWUD (e.g., drug treatment facilities). Indeed, improved collaboration between harm reduction programs and surveillance professionals, including medical examiners and crime laboratory personnel, could circumvent and augment delays in reporting systems. Of course, caution is needed in the development and deployment of warning systems to avoid sensationalizing harms or generating “alert fatigue,” where PWUD and their communities are inundated with information [28, 29]. These findings also emphasize the benefit of embedding research within harm reduction settings to further empower these organizations in responding to emerging challenges.

PWUD demonstrated a predominantly protective approach to xylazine emergence by modifying their drug consumption routes and reducing injection drug use, aiming to mitigate potential harms associated with xylazine adulteration. While often discussed in the context of xylazine here, this echoes a broader literature that reveals an elevated prevalence of smoking among people who previously injected opioids on the West Coast of North America [30–32].

Xylazine use has been associated with severely necrotizing skin infections that produce wounds even distal to the injection site or when individuals are smoking [10, 12, 33]. While more research is needed on the health implications of transitioning away from injection drug use towards other routes of administration (e.g., smoking) and its consequences, it is possible that transitioning away from injecting could reduce the likelihood of tissue necrosis or subsequent SSTIs [34]. However, it is still unclear if transitioning from injecting to smoking opioids (especially those adulterated with xylazine) decreases one’s risk for overdose and what the impacts are on cardiovascular health [35].

While clients’ reliance on alternative consumption routes and peer networks for safety highlights the importance of peer-based harm reduction approaches, increased stimulant use and ‘human testing’ practices, intended to counteract xylazine’s sedative effects and verify drug safety, raises concerns about heightened susceptibility to overdose and other adverse outcomes [3, 36]. Increased polysubstance use with stimulants may increase clients’ overall drug consumption, thereby increasing their risk for additional adverse events (e.g., cardiac arrest, overdose, and SSTIs) [37–39]. Also, reports of using drugs alone underscore the need for additional interventions to address social isolation and enhance safety while people consume unregulated substances. One such intervention that may overcome these challenges could be implementing a phone-based overdose response service, as seen in Canada [40].

The phenomenon of clients seeking out xylazine for its unique effects challenges existing literature on individuals’ preferences for xylazine and has implications for addiction treatment in the context of an evolving drug supply [41–43]. For example, more research is needed to develop guidelines for managing buprenorphine initiation while individuals are using xylazine-adulterated opioids to help mitigate anxiety and xylazine-related withdrawal symptoms [44]. On the other hand, despite efforts to reduce exposure to xylazine, some clients reported increasing their consumption of opioids, driven by the need to counteract diminished opioid availability and withdrawal symptoms associated with xylazine’s short half-life [45]. This finding contradicts previous hypotheses suggesting xylazine’s role in extending the duration of opioids’ effects [12, 46], indicating a nuanced interplay between substance availability, dependence, and desired effects.

Our findings underscore the dynamic nature of PWUD’s responses to xylazine. While some adaptive behaviors aimed to mitigate immediate risks, they may have inadvertently contributed to new challenges, necessitating additional harm reduction strategies, including the rollout of drug-checking services or other advancements (such as safer smoking supplies). However, only fentanyl testing is legal in the state of Florida, and safer smoking supplies are prohibited under the state’s current paraphernalia statute [16]. In the wake of xylazine emergence, other novel psychoactive substances (e.g., nitazenes), and a seemingly national trend of people transitioning from injecting to smoking, further consideration of these punitive laws is necessary to reduce substance-related harm and healthcare costs.

Though our study highlights many important implications for medical and harm reduction providers in the context of rising xylazine adulteration internationally, our findings should be interpreted with some limitations in mind. First, our findings may not be entirely transferable to other sociodemographic communities given our relatively small sample size of primarily White males who use drugs and staff of one SSP in an urban center in the Southern U.S. Second, our interviews took place after the emergence of xylazine in Miami and the implementation of XTS at the SSP, which may have impacted recall. Third, participants were asked to reflect on their experiences using unregulated opioids when describing the emergence of xylazine. While interviews focused on opioid supplies, it is possible that participants may have also recalled experiences using other unregulated drugs adulterated with xylazine (e.g., stimulants). Despite these limitations, our findings may have important implications for regions now seeing xylazine and other novel psychoactive substances (e.g., nitazenes) for the first time, which are increasingly implicated in health consequences for PWUD internationally [4, 47]. Further research is needed to validate and expand upon our findings, particularly in diverse geographic contexts and with larger sample sizes.

## Conclusions

This study sheds light on the unprecedented emergence of xylazine within the unregulated opioid supply, presenting a comprehensive examination of PWUD responses through the lens of SSP staff and client experiences. These findings carry implications for prevention programming in regions encountering xylazine or other novel psychoactive substances such as nitazenes. Through interdisciplinary collaboration and community-based harm reduction efforts, SSP staff can leverage client perspectives to inform rapid responses to emerging adulterants, safeguarding against potential health consequences and promoting safer drug use practices. However, as evidenced by the complex interplay of protective behaviors and unintended harms identified in our study, addressing the evolving challenges posed by xylazine requires ongoing adaptation and innovation in harm reduction strategies. Moving forward, it is imperative to prioritize client feedback, foster interdisciplinary collaboration, and advocate for evidence-based interventions and policies, such as drug checking and safer smoking supplies, that can mitigate the impact of emerging substances on public health.

### Electronic supplementary material

Below is the link to the electronic supplementary material.

Supplementary Material 1

Supplementary Material 2

## Data Availability

Our research data includes sensitive or confidential information and is therefore not available.

## Abbreviations

SSP: Syringe services program
IMF: Illicitly manufactured fentanyl
PWUD: People who use drugs
SSTI: Skin and soft tissue infection
XTS: Xylazine test strip

## References

[1] Giraudon I, Abel-Ollo K, Vanaga-Arāja D, Heudtlass P, Griffiths P. Nitazenes represent a growing threat to public health in Europe. Lancet Public Health. 2024;9(4):e216. 38408464 10.1016/S2468-2667(24)00024-0

[2] Griffiths PN, Seyler T, De Morais JM, Mounteney JE, Sedefov RS. Opioid problems are changing in Europe with worrying signals that synthetic opioids may play a more significant role in the future. Addiction. 2023. 10.1111/add.16420 38129293

[3] Friedman J, Shover CL. Charting the fourth wave: Geographic, temporal, race/ethnicity and demographic trends in polysubstance fentanyl overdose deaths in the United States, 2010–2021. Addiction. 2023;118(12):2477–85. 37705148 10.1111/add.16318

[4] Friedman JR, Commentary on, Copeland, et al. Does xylazine in the United Kingdom foreshadow a European synthetic polysubstance drug crisis? Addiction. 2024;119(7):1310–2. 38570907 10.1111/add.16498

[5] Friedman J, Montero F, Bourgois P, Wahbi R, Dye D, Goodman-Meza D, Shover C. Xylazine spreads across the US: a growing component of the increasingly synthetic and polysubstance overdose crisis. Drug Alcohol Depend. 2022;233:109380. 35247724 10.1016/j.drugalcdep.2022.109380 PMC9128597

[6] Government of Canada. Opioid- and Stimulant-related Harms in Canada. 2024.

[7] Bowles JM, McDonald K, Maghsoudi N, Thompson H, Stefan C, Beriault DR, et al. Xylazine detected in unregulated opioids and drug administration equipment in Toronto, Canada: clinical and social implications. Harm Reduct J. 2021;18(1):104. 34645480 10.1186/s12954-021-00546-9 PMC8511847

[8] Ruiz-Colón K, Chavez-Arias C, Díaz-Alcalá JE, Martínez MA. Xylazine intoxication in humans and its importance as an emerging adulterant in abused drugs: a comprehensive review of the literature. Forensic Sci Int. 2014;240:1–8. 24769343 10.1016/j.forsciint.2014.03.015

[9] Kariisa M, Patel P, Smith H, Bitting J. Notes from the field: xylazine detection and involvement in drug overdose deaths - United States, 2019. MMWR Morb Mortal Wkly Rep. 2021;70(37):1300–2. 34529640 10.15585/mmwr.mm7037a4 PMC8445380

[10] Malayala SV, Papudesi BN, Bobb R, Wimbush A. Xylazine-Induced skin ulcers in a person who injects drugs in Philadelphia, Pennsylvania, USA. Cureus. 2022;14(8):e28160. 36148197 10.7759/cureus.28160 PMC9482722

[11] Soderquist M, Delgado G, Abdelfattah H, Thoder J, Solarz M. Necrotic Upper-Extremity infections in people who inject drugs: a Case Series. J Hand Surg Am. 2023. 10.1016/j.jhsa.2023.04.001 37178065

[12] Reyes JC, Negrón JL, Colón HM, Padilla AM, Millán MY, Matos TD, Robles RR. The Emerging of Xylazine as a New drug of abuse and its Health consequences among drug users in Puerto Rico. J Urb Health. 2012;89(3):519–26. 10.1007/s11524-011-9662-6 PMC3368046 22391983

[13] Johnson J, Pizzicato L, Johnson C, Viner K. Increasing presence of xylazine in heroin and/or fentanyl deaths, Philadelphia, Pennsylvania, 2010–2019. Inj Prev. 2021;27(4):395. 33536231 10.1136/injuryprev-2020-043968

[14] Bradford W, Figgatt M, Scott KS, Marshall S, Eaton EF, Dye DW. Xylazine co-occurrence with illicit fentanyl is a growing threat in the Deep South: a retrospective study of decedent data. Harm Reduct J. 2024;21(1):46. 38378660 10.1186/s12954-024-00959-2 PMC10880285

[15] Potoukian RB, Gonyea J, Shoff EN, Hime GW, Moore DM. Prevalence of xylazine in overdose cases: an analysis of Miami-Dade County medical examiner case data. J Forensic Sci. 2023;68(6):2205–10. 37658657 10.1111/1556-4029.15375

[16] Controlled Substance Testing. Florida House of Representatives. 2023.

[17] Hauschild MH, Warp PV, Tookes HE, Yakir E, Malhotra B, Malik S, et al. Prevalence of xylazine among people who inject drugs seeking medical care at a syringe services program clinic: Miami, Florida, 2023. Drug Alcohol Depend Rep. 2023;9:100209. 38162510 10.1016/j.dadr.2023.100209 PMC10755708

[18] Warp PV, Hauschild M, Tookes HE, Ciraldo K, Serota DP, Cruz I. A Confirmed Case of Xylazine-Induced Skin Ulcers in a Person Who Injects Drugs in Miami, Florida, USA. Res Sq. 2023. 10.1186/s12954-024-00978-z PMC10941577 38491467

[19] Bazzi AR, Valasek CJ, Streuli SA, Vera CF, Harvey-Vera A, Philbin MM, et al. Long-acting Injectable Human Immunodeficiency Virus Pre-exposure Prophylaxis Preferred Over other modalities among people who inject drugs: findings from a qualitative study in California. AIDS Patient Care STDS. 2022;36(7):254–62. 35727647 10.1089/apc.2022.0068 PMC9464050

[20] Valasek CJ, Streuli SA, Pines HA, Mittal ML, Strathdee SA, Vera CF, et al. COVID-19 vaccination acceptability and experiences among people who inject drugs in San Diego County. Prev Med Rep. 2022;30:101989. 36148319 10.1016/j.pmedr.2022.101989 PMC9484100

[21] Quijano T, Crowell J, Eggert K, Clark K, Alexander M, Grau L, Heimer R. Xylazine in the drug supply: emerging threats and lessons learned in areas with high levels of adulteration. Int J Drug Policy. 2023;120:104154. 37574646 10.1016/j.drugpo.2023.104154

[22] Zhu DT, Friedman J, Bourgois P, Montero F, Tamang S. The emerging fentanyl-xylazine syndemic in the USA: challenges and future directions. Lancet. 2023;402(10416):1949–52. 37634523 10.1016/S0140-6736(23)01686-0 PMC10842070

[23] Wong SC, Curtis JA, Wingert WE. Concurrent Detection of Heroin, Fentanyl, and Xylazine in Seven Drug-related deaths reported from the Philadelphia Medical Examiner’s Office. J Forensic Sci. 2008;53(2):495–8. 18284526 10.1111/j.1556-4029.2007.00648.x

[24] Artigiani EE, Wish ED. Introducing the National Drug early warning system. Curr Opin Psychiatry. 2020;33(4):319–25. 32250985 10.1097/YCO.0000000000000610 PMC7386464

[25] Rosenblum D, Unick J, Ciccarone D. The rapidly changing US Illicit Drug Market and the potential for an improved early warning system: evidence from Ohio Drug Crime Labs. Drug Alcohol Depend. 2020;208:107779. 31931266 10.1016/j.drugalcdep.2019.107779 PMC7096152

[26] Park JN, Tardif J, Thompson E, Rosen JG, Lira JAS, Green TC. A survey of north American drug checking services operating in 2022. Int J Drug Policy. 2023;121:104206. 37797571 10.1016/j.drugpo.2023.104206 PMC10843152

[27] Grace Rose C, Pickard AS, Kulbokas V, Hoferka S, Friedman K, Epstein J, Lee TA. A qualitative assessment of key considerations for drug checking service implementation. Harm Reduct J. 2023;20(1):151. 37848875 10.1186/s12954-023-00882-y PMC10580636

[28] Brien R, Volpe I, Grigg J, Lyons T, Hughes C, McKinnon G, et al. Co-designing drug alerts for health and community workers for an emerging early warning system in Victoria, Australia. Harm Reduct J. 2023;20(1):30. 36894933 10.1186/s12954-023-00761-6 PMC9995746

[29] Volpe I, Brien R, Grigg J, Tzanetis S, Crawford S, Lyons T, et al. We don’t live in a harm reduction world, we live in a prohibition world’: tensions arising in the design of drug alerts. Harm Reduct J. 2023;20(1):3. 36624508 10.1186/s12954-022-00716-3 PMC9829230

[30] Eger WH, Abramovitz D, Bazzi AR, Bórquez A, Vera CF, Harvey-Vera A, et al. Changes in injecting versus smoking heroin, fentanyl, and methamphetamine among people who inject drugs in San Diego, California, 2020–2023. Drug Alcohol Depend. 2024;259:111318. 38692135 10.1016/j.drugalcdep.2024.111318 PMC11463215

[31] Kral AH, Lambdin BH, Browne EN, Wenger LD, Bluthenthal RN, Zibbell JE, Davidson PJ. Transition from injecting opioids to smoking fentanyl in San Francisco, California. Drug Alcohol Depend. 2021;227:109003. 34482046 10.1016/j.drugalcdep.2021.109003 PMC10790652

[32] Kamal A, Ferguson M, Xavier JC, Liu L, Graham B, Lock K, Buxton JA. Smoking identified as preferred mode of opioid safe supply use; investigating correlates of smoking preference through a 2021 cross-sectional study in British Columbia. Subst Abuse Treat Prev Policy. 2023;18(1):27. 37194018 10.1186/s13011-023-00515-4 PMC10186314

[33] Wei J, Wachuku C, Berk-Krauss J, Steele KT, Rosenbach M, Messenger E. Severe cutaneous ulcerations secondary to xylazine (tranq): a case series. JAAD Case Rep. 2023;36:89–91. 37274146 10.1016/j.jdcr.2023.04.016 PMC10232457

[34] Megerian CE, Bair L, Smith J, Browne EN, Wenger LD, Guzman L, et al. Health risks associated with smoking versus injecting fentanyl among people who use drugs in California. Drug Alcohol Depend. 2024;255:111053. 38128362 10.1016/j.drugalcdep.2023.111053

[35] Koslik HJ, Joshua J, Cuevas-Mota J, Goba D, Oren E, Alcaraz JE, Garfein RS. Prevalence and correlates of obstructive lung disease among people who inject drugs, San Diego, California. Drug Alcohol Depend. 2020;214:108158. 32652379 10.1016/j.drugalcdep.2020.108158 PMC7331511

[36] Ciccarone D. The rise of illicit fentanyls, stimulants and the fourth wave of the opioid overdose crisis. Curr Opin Psychiatry. 2021;34(4):344–50. 33965972 10.1097/YCO.0000000000000717 PMC8154745

[37] Serota DP, Bartholomew TS, Tookes HE. Evaluating differences in Opioid and Stimulant Use-associated Infectious Disease hospitalizations in Florida, 2016–2017. Clin Infect Dis. 2021;73(7):e1649–57. 32886747 10.1093/cid/ciaa1278 PMC8492144

[38] Cahn BA, Bartholomew TS, Patel HP, Pastar I, Tookes HE, Lev-Tov H. Correlates of injection-related wounds and skin infections amongst persons who inject drugs and use a syringe service programme: a single center study. Int Wound J. 2021;18(5):701–7. 33586860 10.1111/iwj.13572 PMC8450795

[39] Friedman J, Shover CL. Charting the fourth wave: Geographic, temporal, race/ethnicity and demographic trends in polysubstance fentanyl overdose deaths in the United States, 2010–2021. Addiction. 2023;n/a(n/a). 10.1111/add.16318 37705148

[40] Rioux W, Teare A, Rider N, Jones S, Ghosh SM. Preference for hotline versus mobile application/countdown-based mobile overdose response services: a qualitative study. Harm Reduct J. 2024;21(1):31. 38317194 10.1186/s12954-024-00944-9 PMC10840257

[41] Hochheimer M, Strickland JC, Rabinowitz JA, Ellis JD, Dunn KE, Huhn AS. Knowledge, preference, and adverse effects of Xylazine among adults in Substance Use Treatment. JAMA Netw Open. 2024;7(2):e240572. 38416493 10.1001/jamanetworkopen.2024.0572 PMC10902730

[42] Collins AB, Macon EC, Levin S, Wunsch C, Wightman RS. It gets you high as a kite but not unsick: characterizations of and responses to a changing local drug supply by people who use drugs in Rhode Island. Int J Drug Policy. 2024;127:104391. 38490014 10.1016/j.drugpo.2024.104391 PMC11127783

[43] Ehrman-Dupre R, Kaigh C, Salzman M, Haroz R, Peterson L-K, Schmidt R. Management of Xylazine Withdrawal in a hospitalized patient: a Case Report. J Addict Med. 2022;16(5). 10.1097/ADM.0000000000000955 35020700

[44] Amin-Esmaeili M, Farokhnia M, Susukida R, Leggio L, Johnson RM, Crum RM, Mojtabai R. Reduced drug use as an alternative valid outcome in individuals with stimulant use disorders: findings from 13 multisite randomized clinical trials. Addiction. 2024;119(5):833–43. 38197836 10.1111/add.16409 PMC11009085

[45] FDA warns about. the risk of xylazine exposure in humans [press release]. November 8, 2022. 2022.

[46] Torruella RA. Xylazine (veterinary sedative) use in Puerto Rico. Subst Abuse Treat Prev Policy. 2011;6(1):7. 21481268 10.1186/1747-597X-6-7 PMC3080818

[47] Galust H, Seltzer JA, Hardin JR, Friedman NA, Salamat J, Clark RF, Harmon J. Adulterants present in the San Diego county fentanyl supply: a laboratory analysis of seized law enforcement samples. BMC Public Health. 2024;24(1):923. 38553721 10.1186/s12889-024-18459-0 PMC10981322

