# Supplementary material for: Syringe services program staff and participant perspectives on changing drug consumption behaviors in response to xylazine adulteration
Source: Harm Reduct J. 2024 Aug 30;21:162. doi: 10.1186/s12954-024-01082-y (PMC11363649; doi:10.1186/s12954-024-01082-y)
Supplement: Supplementary file 1 — Supplementary Material 1 [file 12954_2024_1082_MOESM1_ESM.docx]

**APPENDIX 1: MIAMI-DADE XYLAZINE QUALITATIVE STUDY: INTERVIEW GUIDE FOR PWID – 06/23/2023**

**PREAMBLE: [READ ALOUD]:** Hi, my name is **[interviewer]** and I’m an interviewer for this project. We’re trying to learn more about ways to help support syringe service programs (SSPs) in providing drug checking and xylazine- (or “tranq”) related services. I would also like to audio-record this interview, with your permission, so I don’t have to take too many notes and can focus on our discussion. We can stop this recording at any time, and all or part of the recording can be deleted at your request. After we transcribe (write up) these recordings and make sure the write-ups are accurate and de-identified, we will destroy the recordings. **Do you have any questions before we begin?** **And to confirm, is it OK with you that I record this interview?**

**[TURN ON RECORDER; READ]:** This is **[interviewer initials: ____________ ]** with **[IDEA ID # _______________ ]** on **[date: ________ ]** at **[time: ____________ ].**

**Can you walk me through when and how you first learned of xylazine (or “tranq”, “tranq-dope”)?**

- Can you recall when you first heard of tranq?

**What can you tell me about tranq? *(Either from personal experience or what you heard from others)***

- What have you heard (or experienced) about how tranq makes you feel?

**How do you feel about tranq?**

- What are some of your biggest concerns regarding tranq?

**Walk me through the last time you tested your drugs.**

- When was the last time you tested your drugs? Why did you decide to test them?
- What has your experience with testing your drugs been like in the past?

**What is your preferred method for testing drugs? For example, before you use, after, using fentanyl test strips, taking a small hit, etc.**

- If our program was to hand out xylazine (or “tranq”) test strips [SHOW PARTICIPANT] - tests similar to fentanyl test strips - would you use them?
- If they were made available to you, about how often would you use xylazine test strips?

**When might xylazine test strips be useful for you? For example, if your dealer changes, if your drugs look different, if word on the street or in the exchange is talking about something new.**

- What might be some barriers that you would face accessing xylazine test strips?

**If you did test your sample and it tested positive for tranq, what would you do with that information?**

- For example: do nothing differently, do a tester hit, throw out your drugs, go to a different dealer in the future, go to that specific dealer in the future, smoke the drugs instead of inject them, sell them to someone who wants tranq, use them as planned without any changes.

**What type of drugs would you want to test with your xylazine test strips? (*Would you want to test all of your drugs?*)**

**[SHOW THEM A PICTURE OF AN FTIR MACHINE AND HOW IT WORKS] If our program was to start checking people’s drugs using a machine that can tell you the specific drugs AND the concentration levels of your sample ONSITE would you use it?****Before answering, note that this machine would require you to bring in a baggie with some drug residue on it or a small sample of your drugs, but is both more accurate and can provide more detail than testing strips**

- Why would you or would you not use this service?
- What would you do with the information it provides to you? For example, if it told you that your dope had tranq in it.
- How would you go about using this type of service? For example, would you be able to bring in your drugs before you use them? Would you come after using to just check what your dealer has been giving you?)
- Would this service be helpful to you?
- What about for other people you know?
- When might you use this type of service? For example, if your dealer changes, if your drugs look different, if word on the street or in the exchange is talking about something new.

**What might be some barriers that you would face accessing a service like this?**

- For example: having to bring part of your drugs or a baggie with you to the needle exchange; sense of urgency in using your supply (because of withdrawal, or concerns about police finding you with it, etc.)

**Have you noticed changes to your drug use or that people you know were changing their drug use since people started talking about tranq?**

- Are you or people you know consuming drugs differently? For example, smoking instead of injecting.
- Are you or people you know seeking out tranq? Trying to avoid it?

**CLOSING**

OK, I believe those are all of my specific questions. **Reflecting on our discussion, is there anything else we haven’t talked about today that you think I should know, especially regarding tranq?**

Please give me just a moment to check to make sure I covered everything. **[CHECK OVER INTERVIEW GUIDE]**. OK, this concludes the interview. **[TURN OFF TAPE RECORDER]** Thank you again for taking time out of your schedule to provide us with this information.
