# Supplementary material for: Syringe services program staff and participant perspectives on changing drug consumption behaviors in response to xylazine adulteration
Source: Harm Reduct J. 2024 Aug 30;21:162. doi: 10.1186/s12954-024-01082-y (PMC11363649; doi:10.1186/s12954-024-01082-y)
Supplement: Supplementary file 2 — Supplementary Material 2 [file 12954_2024_1082_MOESM2_ESM.docx]

**APPENDIX 2: MIAMI-DADE XYLAZINE QUALITATIVE STUDY: INTERVIEW GUIDE FOR KEY INFORMANTS – 06/23/202**

**PREAMBLE: [READ ALOUD]:** Hi, my name is **[interviewer]** and I’m an interviewer for this project. We’re trying to learn more about ways to help support syringe service programs (SSPs) in providing drug checking and xylazine- (or “tranq”) related services. I would also like to audio-record this interview, with your permission, so I don’t have to take too many notes and can focus on our discussion. We can stop this recording at any time, and all or part of the recording can be deleted at your request. We will transcribe recordings for text analysis and destroy recordings after verifying that transcripts are accurate and de-identified. **Do you have any questions before we begin?** **And to confirm, is it OK with you that I record this interview?**

**[TURN ON RECORDER; READ]:** This is **[interviewer initials: ____________ ]** with **[participant initials:______________]** on **[date: ________ ]** at **[time: ____________ ].**

**Can you tell me a little bit about what you do?**

- What type of organization do you work for?

**When did you first start working with people who use drugs?**

- About how long ago?

**Can you walk me through when and how you first learned of xylazine (or “tranq”)?**

- Can you recall when you first heard of xylazine?

**What can you tell me about xylazine?**

- What have you heard about xylazine’s effects? Both how it makes people feel and its positive or negative consequences.
- What are some of your biggest concerns regarding xylazine?

**What type of drug checking services does your organization offer?**

- For example, fentanyl test strips; xylazine test strips; FTIR; urine testing.

**Walk me through the last time you tested someone’s drugs or did a urine drug screen.**

- For example, using fentanyl test strips.
- What impact do you think this had on the participant?

**What are some of the benefits of drug testing?**

- For participants? For the organization?
- If your organization was to hand out xylazine test strips [SHOW PARTICIPANT] – tests similar to fentanyl test strips – do you think they would be useful for people?

**If your organization started offering xylazine test strips, where and when would you offer them to a participant?**

- What types of drugs would you want people to test with their xylazine test strips?
- How would you prioritize who you distribute xylazine test strips to?

**What might be some barriers to implementing xylazine test strips at your organization?**

- How would you try to overcome those barriers?

**[SHOW THEM A PICTURE OF AN FTIR MACHINE AND HOW IT WORKS] If your organization was to implement drug checking using a machine that can tell someone the specific drugs AND the concentration levels of their sample ONSITE would it be useful?**

**Before answering, note that this machine would require someone to bring in a baggie with some drug residue on it or a small sample, but this method is both more accurate and can provide more detail than testing strips**

- Why would it be useful? For participants? For the organization?
- What do you see as the major differences between drug checking ONSITE using a machine over test strips that could be given out/distributed?
- What might be some downsides of using this technology?
- What are some barriers to implementing a drug checking machine at your organization?

**If your organization could send a drug sample, or residue from a baggie, to an EXTERNAL organization for testing that can tell you the sample’s specific contents AND concentration – do you think that service would be useful?**

- What do you see as the major differences using an EXTERNAL lab versus onsite drug testing with a machine?
- With a xylazine test strip?

**What would you do if someone’s sample or urine was reactive for xylazine?**

- What would you do with the information it provides?
- For example, tell them how to inject safely; encourage them to switch to smoking drug use; encourage them to continue using test strips or drug checking; inform them of the differences between xylazine-related overdoses and opioid-overdoses, etc.

**Have you noticed changes to your participant or patient’s drug use since people started talking about xylazine?**

- Are people using more of certain types of drugs now?
- Are people consuming drugs differently? For example, smoking instead of injecting.
- Are you hearing reports of people seeking out xylazine? Trying to avoid it?

**CLOSING**

OK, I believe those are all of my specific questions. **Reflecting on our discussion, is there anything else we haven’t talked about today that you think I should know, especially regarding xylazine?**

Please give me just a moment to check to make sure I covered everything. **[CHECK OVER INTERVIEW GUIDE]**. OK, this concludes the interview. **[TURN OFF TAPE RECORDER]**

Thank you again for taking time out of your schedule to provide us with this information.
